# Evaluation of Copper Incorporation into Titanium via Ion Plating Diversified: Morphological, Structural, and Preliminary Biological Assessment

**DOI:** 10.1021/acsomega.5c09662

**Published:** 2026-01-30

**Authors:** Eduardo Antônio Zanella, Camila Baldasso, Cesar Aguzzoli, Wendel Paulo Silvestre

**Affiliations:** † Postgraduate Program in Process Engineering and Technologies (PGEPROTEC), 58802 Universidade de Caxias Do Sul, Caxias Do Sul, RS 95070-560, Brazil; ‡ Postgraduate Program in Materials Science and Engineering (PPGMAT), 58802 Universidade de Caxias Do Sul, Caxias Do Sul, RS 95070-560, Brazil

## Abstract

Surface contamination of titanium-based implants by microorganisms remains a major clinical challenge. In this study, grade 1 titanium substrates were modified by low-energy ion plating diversified (IPD) with copper ions at 3 keV and 7 keV to evaluate their morphological, structural, and preliminary biological behavior. Characterization by SEM, EDS, and XRF confirmed homogeneous copper incorporation, with similar concentrations (∼0.3 wt %) for both energies. SRIM simulations indicated Gaussian implantation profiles with peak concentrations at 4 nm and 6 nm below the surface, respectively. Salt spray testing showed that copper implantation did not impair the corrosion resistance of titanium. However, biological assays using *Staphylococcus aureus* revealed no inhibitory halos or significant cell death, indicating that copper ions implanted at these energies do not reach the surface or diffuse into the medium. These findings define the physical limitations of low-energy IPD for antimicrobial surface design, providing helpful guidance for future process optimization.

## Graphical abstract

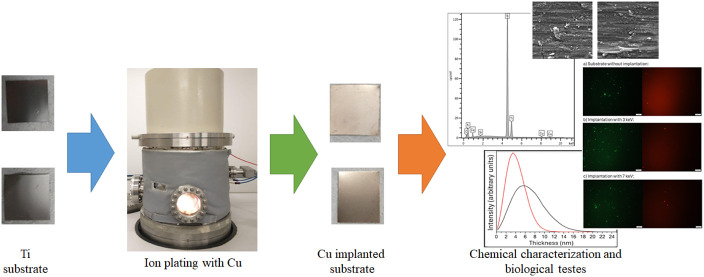

## Introduction

Agrawal et al. define biomaterial as any surface or structural material capable of interacting with biological systems.^1^ Farag complements this concept by bringing the definition given by the National Health Institution, which states that “It is any natural or synthetic substance or combination of both—except medicines—that can be used to replace or restore, totally or partially, tissues, organs, or functions of the body, to maintain or improve the individual’s quality of life”. A wide range of materials can serve this purpose, including metals, ceramics, glass, and others.^2^ Biomaterials can be utilized in various applications, including cardiac repairs,^3^ bone tissue defect repairs,^4^ orthopedics,^5^ orthodontics,^6^ and pharmaceutical applications.^3^

The field of biomaterials is multidisciplinary, integrating knowledge from medicine, biology, chemistry, physics, and materials science.^4^ Among the primary characteristics required of a biomaterial are resistance to corrosion and wear, which are essential factors for ensuring durability and safety in medical applications. Furthermore, these materials must exhibit high mechanical strength, low stiffness (reduced Young’s modulus), good adhesion, adequate biological functionality, the absence of toxicity, and, above all, excellent biocompatibility. Biocompatibility is a fundamental requirement, as it refers to the material’s ability to interact safely and effectively with the organism, eliciting an appropriate host response in a given clinical application.^5^

Titanium has been widely used in biomedical applications since the 1950s. This metal, as well as its alloys, stands out for its excellent mechanical and tribological properties, high corrosion resistance, and biocompatibility. Furthermore, titanium has the remarkable ability to integrate directly with living bone at the implant site, promoting osseointegration without the need for additional fixation agents.^6^ This combination of biocompatibility and mechanical performance contributes to its prominence as one of the most effective materials in implant manufacturing.^7^

Despite advances in biomaterials and sterilization techniques, biomaterial-associated infections (BAIs) remain a significant challenge in clinical practice. They occur when bacteria adhere to the surface of the material and form biofilms—structures that are highly resistant to the immune system and conventional antibiotics—making them difficult to control.^8,9^ BAIs are common postoperative complications that can lead to implant failure, prolonged hospital stays, increased morbidity, and high costs. Furthermore, biomaterials can facilitate the entry of microorganisms into the body, thereby increasing the risk of infection. The use of antibiotics is not always effective against biofilms and can aggravate antimicrobial resistance to these drugs.^9^

Given this scenario, it is crucial to develop effective strategies to mitigate the risk of implant-related infections and their associated complications. Among the available alternatives is the use of material surface modification techniques to functionalize the implant surface, giving it antimicrobial properties.^10^ Some of the techniques that can be employed to alter the surface (or the region close to it) include plasma treatments, the insertion of thin films, and ion implantation.^11^

Ion implantation is a physical surface modification technique that involves accelerating ions of a specific element, allowing them to penetrate the substrate. Performed at low temperatures, the process has a penetration spectrum of approximately 1 μm.^12^ Its primary disadvantage is the high energy required, which can range from 100 keV to 2 MeV. Alternatively, the ion plating technique can be used, in which the sample is subjected to a bombardment (continuous or periodic) of energized particles, capable of promoting changes in the material’s properties.^13^ Studies conducted by Souza et al. demonstrated the effectiveness of this method when using low energy levels, 2 keV and 4 keV, to modify the titanium surface with silver.^14^

Among the primary metals used in surface treatments for antimicrobial purposes, copper and silver are the most notable. Both are highly effective in inactivating bacteria, fungi, and viruses, and are widely used in the modification of biomaterials as well as in clinical and hospital environments.^15^ Coatings applied through techniques such as ion implantation have shown promising results in incorporating these metals into the substrate, promoting effective antimicrobial properties with low cytotoxicity.^16^ Although silver is recognized for its strong bactericidal activity, copper also performs excellently, especially in combating viruses,^15^ and has the added advantage of being less expensive, which makes it a viable alternative for large-scale applications.^17^

Thus, copper stands out as a broad-spectrum antimicrobial agent, with practical applications against various microorganisms, especially when used in the form of nanoparticles,^18^ coatings,^19^ or metallic surfaces.^15^ In the studies by Tripathi et al., the effectiveness of copper against both Gram-negative and Gram-positive bacteria has been demonstrated. The authors coated nanotextured stainless steel surfaces with copper via electrochemical deposition and achieved a ∼97% reduction in *Escherichia coli* (Gram-negative) and a ∼99% reduction in *Staphylococcus epidermidis* within 30 min of contact.^20^ The antifungal properties of copper were demonstrated by Padmavathi et al., who synthesized CuO and Cu_2_O nanoparticles and proved the material’s effectiveness against the fungus *Candida albicans*.^21^ Against viruses, Behzadinasab et al. coated various surfaces with Cu_2_O particles embedded in a polyurethane matrix to evaluate the reduction in the SARS-CoV-2 viral load. After 1 h of exposure, an average reduction of approximately 99.9% in viral load was observed compared to uncoated surfaces.^22^ These results indicate that copper acts as a broad-spectrum agent with rapid, long-lasting, and safe action against viruses, bacteria, and fungi. Therefore, copper has been confirmed as an excellent option for antimicrobial applications in various fields.

Although numerous studies have demonstrated the antimicrobial effectiveness of copper when used as a surface coating on titanium, there is still a limited understanding of how copper behaves when implanted into the titanium matrix itself. Unlike coating processes, ion implantation embeds copper atoms below the surface, potentially altering not only the antimicrobial potential but also the physicochemical properties of the material.^23^ Therefore, this work focuses on evaluating the morphological, structural, and corrosion resistance properties of titanium modified by copper ion implantation via the Ion Plating Diversified (IPD) technique at low energies (3 keV and 7 keV), while also performing preliminary antimicrobial assays to explore the biological response of the implanted surfaces.

The central hypotheses of this study are that (i) copper can be successfully incorporated into titanium using IPD without compromising its structural integrity or corrosion resistance; (ii) implantation energy directly influences the depth and distribution of copper ions; and (iii) the resulting subsurface copper profile may limit bacterial inhibition, thus defining the physical constraints of low-energy IPD for future antimicrobial surface development.

## Materials and Methods

The commercially pure titanium (cp Ti) substrate used to carry out the project was obtained from a company authorized to manufacture orthopedic and dental implants, Sandinox Comércio, Importação e Exportação Ltda (Sorocaba—SP). The specimens were cut into squares measuring 20 mm × 20 mm and 0.3 mm thick.

The material specifications followed a standard chemical composition established by the ASTM F67 grade 1 standard, with 0.03 wt % N, 0.08 wt % C, 0.015 wt % H, 0.20 wt % Fe, and 0.18 wt % O, corresponding to a titanium (Ti) purity of 99.675%, certified by the manufacturer.

The copper used for evaporation and IPD was supplied by Kurt J. Lesker Company in the form of pellets with a purity of 99.99%.

### Sample Preparation and Implantation of Copper Ions at Low Energy

To perform surface cleaning, the titanium plates were initially cleaned with cotton wool and analytical-grade acetone to remove coarse dirt. Then, the samples were subjected to an ultrasonic bath for 20 min. Afterward, the plates were dried with a hot air dryer and placed in a vacuum chamber for the proposed surface treatment.

The low-energy ion implantation process was conducted using Ion Plating Diversified (IPD) equipment. This equipment consists of a vacuum chamber built and adapted by the Surface Engineering and Thermal Treatment Laboratory at the University of Caxias do Sul, with a diameter of 600 mm and a height of 900 mm.

The equipment’s sample holder could hold up to 30 samples. The system was evacuated until a low pressure was achieved, about 1 × 10^–7^ mbar. The chamber ventilation and sample cooling were performed using commercial-grade, pure nitrogen gas.

To carry out the implantation, the polarization energy parameter (BIAS) was adjusted to 3 keV and 7 keV to investigate how ion energy affects copper incorporation behavior in titanium. These values were selected to provide distinct implantation energies within the low-energy regime, allowing for a comparison of the copper penetration and distribution profiles. All other process parameters were kept constant to ensure that any observed differences could be attributed solely to the applied ion energy. The implantation process was conducted under a source voltage of 6 kV, an emission current of 50 mA, and a filament current of 16 A, with polarization energies of 3 keV and 7 keV, respectively.

As the sample holder held up to 30 plates, the implantation process was carried out in two batches, containing 60 samples. The first batch was conducted with a polarization energy of 3 keV, while the second batch was conducted at 7 keV, ensuring better uniformity between the samples within each batch. A quartz microbalance was also used to monitor the amount of copper deposited on the samples, ensuring better uniformity.

### Physicochemical Characterization

The morphology of the samples was examined using Scanning Electron Microscopy with a Field Emission Gun (SEM-FEG). At the same time, the presence of copper implantation was verified by energy-dispersive X-ray spectroscopy (EDS). The total amount of copper incorporated into the titanium surface was quantified by using X-ray fluorescence (XRF). Additionally, ion trajectories and energy losses were simulated by using the Stopping and Range of Ions in Matter (SRIM) program to estimate the concentration and depth profile of copper ions at various implantation energies.

SEM-FEG and EDS analyses were carried out at the Central Microscopy Laboratory of the University of Caxias do Sul (UCS) using a high-performance scanning microscope (Tescan MIRA 3). SEM provided high-resolution images of the titanium surface morphology, whereas EDS enabled chemical characterization of the samples and identification of the elements present. XRF measurements were performed by using a Fischer X-ray fluorescence spectrometer at Mundial S.A., allowing for the quantitative determination of the copper content in each sample.

The SRIM simulations were performed to model the behavior of copper ions under low-energy acceleration conditions (3 keV and 7 keV), providing estimates of the ion penetration depth and distribution.

### Corrosion Resistance Assay

The samples were subjected to a salt spray test to evaluate corrosion resistance. The test was conducted in a Bass Equipamentos LTDA salt-mist chamber (model USC) at Mundial S.A. Six samples from each batch were placed in the chamber, with both the front and back faces exposed to the saline mist, ensuring uniform exposure on all faces. Pure titanium- and copper-implanted samples were compared to assess the influence of surface modification on corrosion resistance. The test was performed for 30 days with daily visual inspections. The saline vapor consisted of a 5 wt % NaCl solution, and the chamber conditions were maintained at a temperature of 35 ± 2 °C, solution pH between 6.5 and 7.2, and pressure of 0.7–1.7 kgf·cm^–2^.

In addition to visual inspection, microscopic analysis was performed using a Pantec optical microscope (Mundial S.A.) to compare the surface conditions of the samples before and after salt exposure, and to detect any microstructural changes caused by corrosion.

### Biological Tests

The biological assays were carried out at the Biotechnology Institute of the University of Caxias do Sul (UCS) using the *Staphylococcus aureus* ATCC 25923 strain. This microorganism was chosen because the species exhibits high virulence and is used in tests, such as those outlined in ISO 22196-2007, as a microorganism to evaluate the measurement of antibacterial activity.^24^ The frozen *S. aureus* strain was thawed and inoculated into liquid Tryptic Soy Broth (TSB) medium, followed by incubation at 37 °C for 24 h. Subsequently, the bacteria were transferred to Petri dishes containing 2 wt % solid TSB medium and incubated under the same conditions to obtain isolated colonies for testing.

#### Qualitative Antibacterial Test

The assay followed a procedure adapted from the CLSI M2-A8 standard.^25^ Three colonies of *S. aureus* were cultured in liquid Luria–Bertani (LB) medium and adjusted using a spectrophotometer (SpectraMax M2e, Molecular Devices) to an optical density (OD600) of 0.1, corresponding to approximately 1 × 10^8^ CFU·mL^–1^. The test materials were previously sterilized in an autoclave at 120 °C for 15 min and dried at room temperature. Petri dishes containing 2 wt % TSB agar were inoculated with 100 μL of the bacterial suspension, which was spread evenly using a Drigalski loop. Each sample was placed with the copper-implanted face in direct contact with the agar surface, and all conditions were tested in duplicate. The plates were incubated at 37 °C for 24 h, after which the bacterial growth inhibition zones were evaluated.

#### Quantitative Antibacterial Test

Quantitative analysis was based on the method adapted from Stiefel et al.^26^ Three *S. aureus* colonies were cultured in liquid LB medium and adjusted to OD600 = 0.1 (≈1 × 10^8^ CFU·mL^–1^), followed by dilution to approximately 1 × 10^5^ CFU·mL^–1^. The test was conducted in a 12-well plate, where the materials (Ti, 3 keV, and 7 keV samples) were immersed in 2 mL of the bacterial suspension and incubated for 24 h at 37 °C. Subsequently, 200 μL of each sample were transferred to a 96-well plate and analyzed by optical density at 600 nm (OD600) using a SpectraMax M2e spectrophotometer to determine bacterial growth.

To assess bacterial viability, the LIVE/DEAD BacLight Bacterial Viability Kit (Invitrogen, Thermo Fisher Scientific) was used according to the manufacturer’s instructions. For staining, 0.2 μL of the fluorescent dye was added to 200 μL of each bacterial suspension and incubated for 15 min, protected from light. Then, 5 μL of each stained sample was mounted on a microscope slide and examined under a fluorescence microscope (Olympus BX43). Live (green) and dead (red) cells were qualitatively evaluated to confirm bacterial viability and membrane integrity.

## Results and Discussion

### Qualitative Elementary Chemical Analysis

The micrographs obtained through Scanning Electron Microscopy (SEM-FEG) are presented in Figure 1, in which (a) refers to the pure substrate, (b) refers to the substrate implanted with 3 keV, and (c) refers to that with 7 keV. The irregularities shown in the analysis are expected for titanium substrates. The images obtained from the implanted samples resemble the micrographs of a titanium surface without implants. It was not possible to observe copper on the surface of the substrate, indicating that the material was implanted in the interstices of the substrate rather than on its surface. This behavior is consistent with the expected ion implantation profile and will be further confirmed by the SRIM simulations presented in the following section.

**Figure 1:**
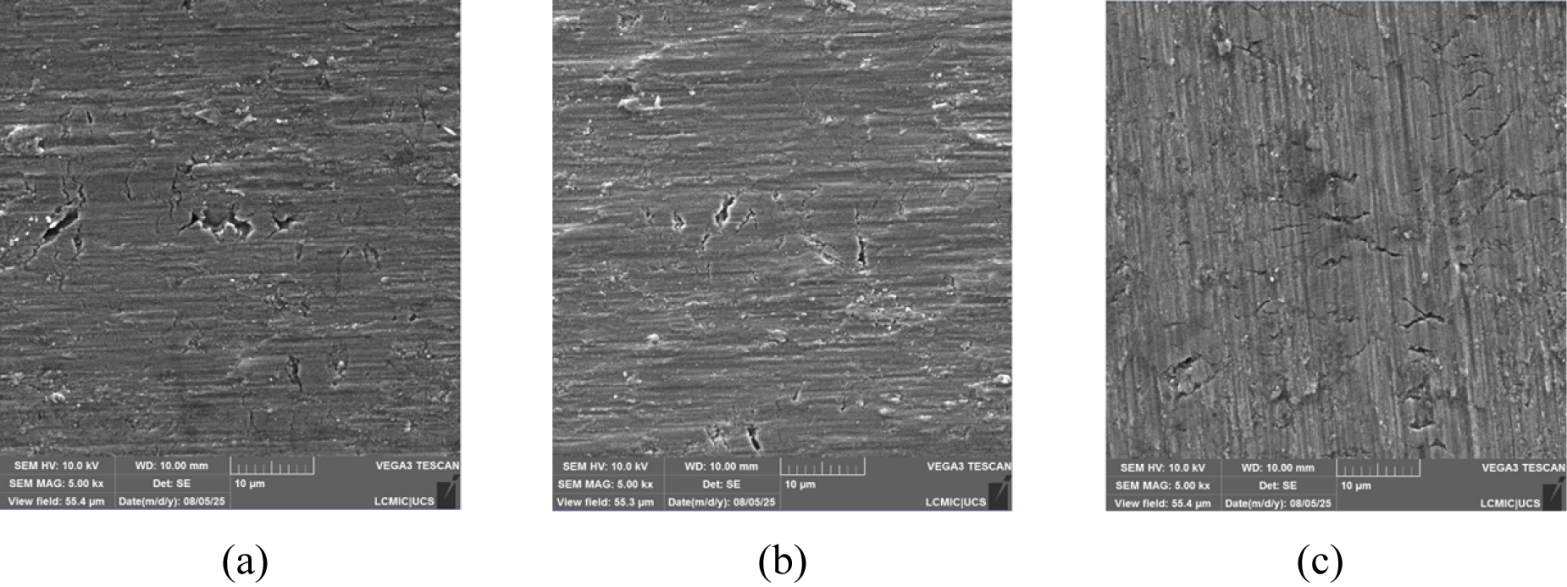
Micrographs of (a) the pure substrate, (b) the substrate implanted with 3 keV, and (c) the substrate implanted with 7 keV, obtained by SEM-FEG.

The analysis of the elemental composition of the samples is shown in Figure 2. The results showed intense titanium peaks and light copper peaks, proving their implantation. It is also possible to notice a carbon peak for both samples. Its presence may be related to the carbon in the crucible used during implantation.

**Figure 2:**
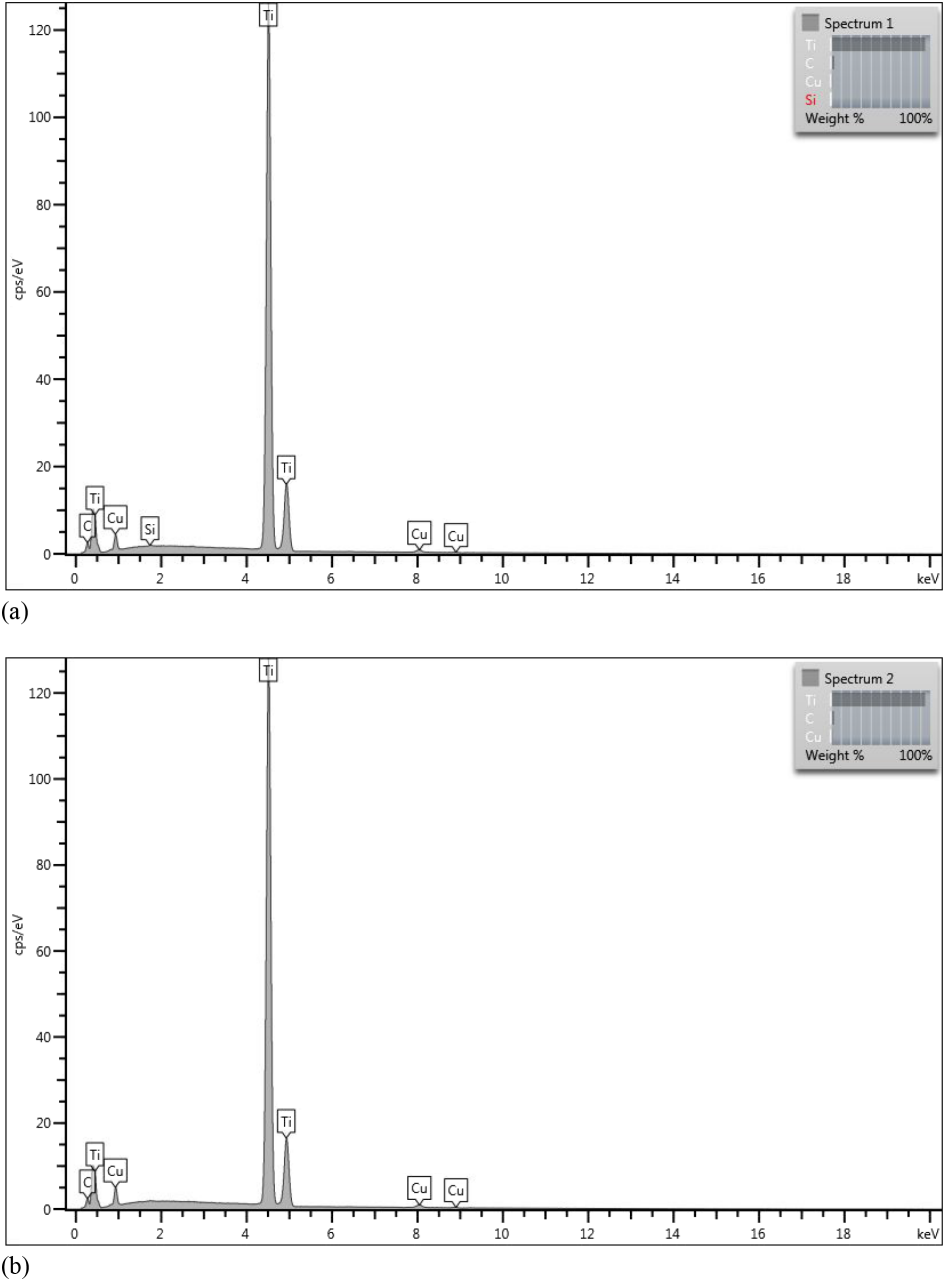
Elemental composition obtained by EDS for implantation energies of (a) 3 keV and (b) 7 keV.

The XRF equipment provided the amount of copper (in percentages) present in each sample. The results obtained were 0.28% (w/w) in the sample where a 3 keV implantation energy was used and 0.29% for 7 keV, with an error associated with the equipment of approximately 5%. This result can be explained by the limited ion implantation efficiency of the IPD process at low energies. Under these conditions, copper ions rapidly reach saturation within the near-surface region, and increasing the energy from 3 to 7 keV primarily affects the projected penetration depth rather than the total incorporated mass. Similar behavior has been reported for other metallic ion implantations performed in the same energy range, where saturation effects occur due to low diffusion and surface scattering phenomena.^27,28^ Furthermore, it was shown that the sample meets the specifications of ASTM F67, not exceeding the maximum limit of contaminants (N, C, H, Fe, and O).

### Depth Simulation

The implantation of copper ions into titanium occurs gradually as these ions lose their energy, either through collisions with the substrate’s atoms or through interactions between the accelerated ions and the electronic orbitals of the target material’s atoms.^29^ The results obtained from the SRIM simulation are shown in Figure 3.

**Figure 3:**
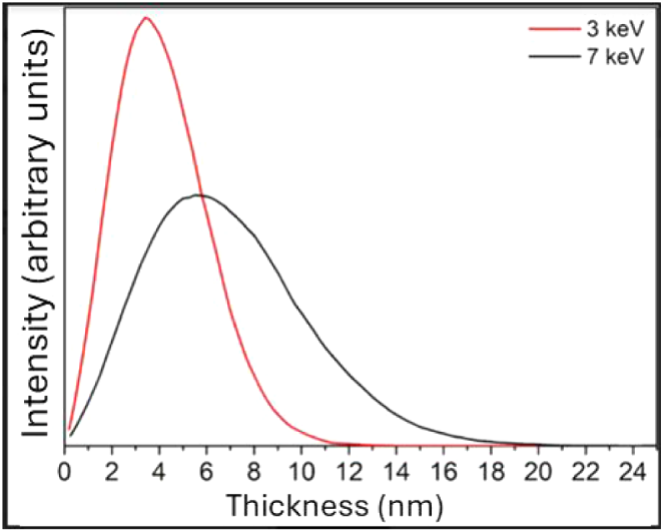
SRIM simulation of the concentration/depth profile.

It is clear that the implantation method induces modifications up to approximately 15–20 nm below the material’s surface. For an energy of 3 keV, the highest concentration of Cu^+^ ions occurred at approximately 4 nm below the surface, while for 7 keV, this peak occurred at 6 nm. Both curves exhibit similar implantation profiles following a Gaussian distribution.

These profiles align well with expectations: at higher polarization energy (7 keV), copper ions are driven deeper into the substrate, whereas at lower implantation energy (3 keV), the maximum concentration occurs closer to the surface.^30,31^ Despite this shift in depth, the total copper concentration measured by XRF remained nearly constant (∼0.3 wt % for both conditions). This consistency suggests that the implantation energy in the range studied primarily affects the penetration depth and distribution of ions, rather than the total implanted mass—a behavior also reported in other low-energy ion implantation studies on titanium.^14^

Furthermore, the Scanning Electron Microscopy analyses did not reveal visible copper deposits on the surface, which supports the assumption that Cu atoms are embedded beneath the outermost layer of the titanium substrate. In contrast, the X-ray Fluorescence measurements indicated a small but measurable copper mass fraction, consistent with the presence of Cu in the bulk of the implanted region rather than at the outermost surface. Therefore, the combination of (i) shallow implantation profiles predicted by SRIM, (ii) absence of surface Cu features in SEM/EDS, and (iii) low yet detectable Cu content in XRF analysis provides a coherent picture of a nonsuperficial, shallow implantation process. This convergence between simulation and experimental characterization strongly supports the interpretation that copper was successfully implanted beneath the titanium surface rather than forming a continuous or segregated surface film.

### Corrosion Resistance Analysis of the Material

The samples were kept in a salt spray chamber for one month, during which they were analyzed daily, except on weekends. It was not possible to observe any visual changes during the period, which indicates that there was no onset of corrosion in the samples, both in the part composed of titanium and in the part implanted with copper.

Although the Cu-implanted samples exhibited a slight copper-like coloration, this appearance does not indicate the formation of a continuous metallic copper film. Ion implantation modifies the native TiO_2_ near-surface layer (defect generation, oxide stoichiometry changes, and associated optical-constant variations), which can produce interference and reflectivity changes and thus alter perceived color even at low net Cu concentrations.^32,33^

Figure 4 shows some samples before being subjected to the salt spray test (a) and the same samples after one month of exposure to the sodium chloride atmosphere (b).

**Figure 4:**
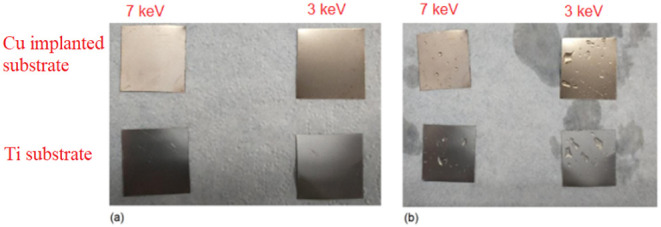
Samples before (a) and after (b) one month of exposure to salt spray.

In addition to the visual aspect, the samples were also analyzed microscopically to assess whether there were any changes at a microscopic level, such as changes in porosity or the presence of early corrosion. No meaningful changes were observed in the substrate’s state before and after it was subjected to the salt spray test. Some of the results obtained are shown in Figure 5.

**Figure 5:**
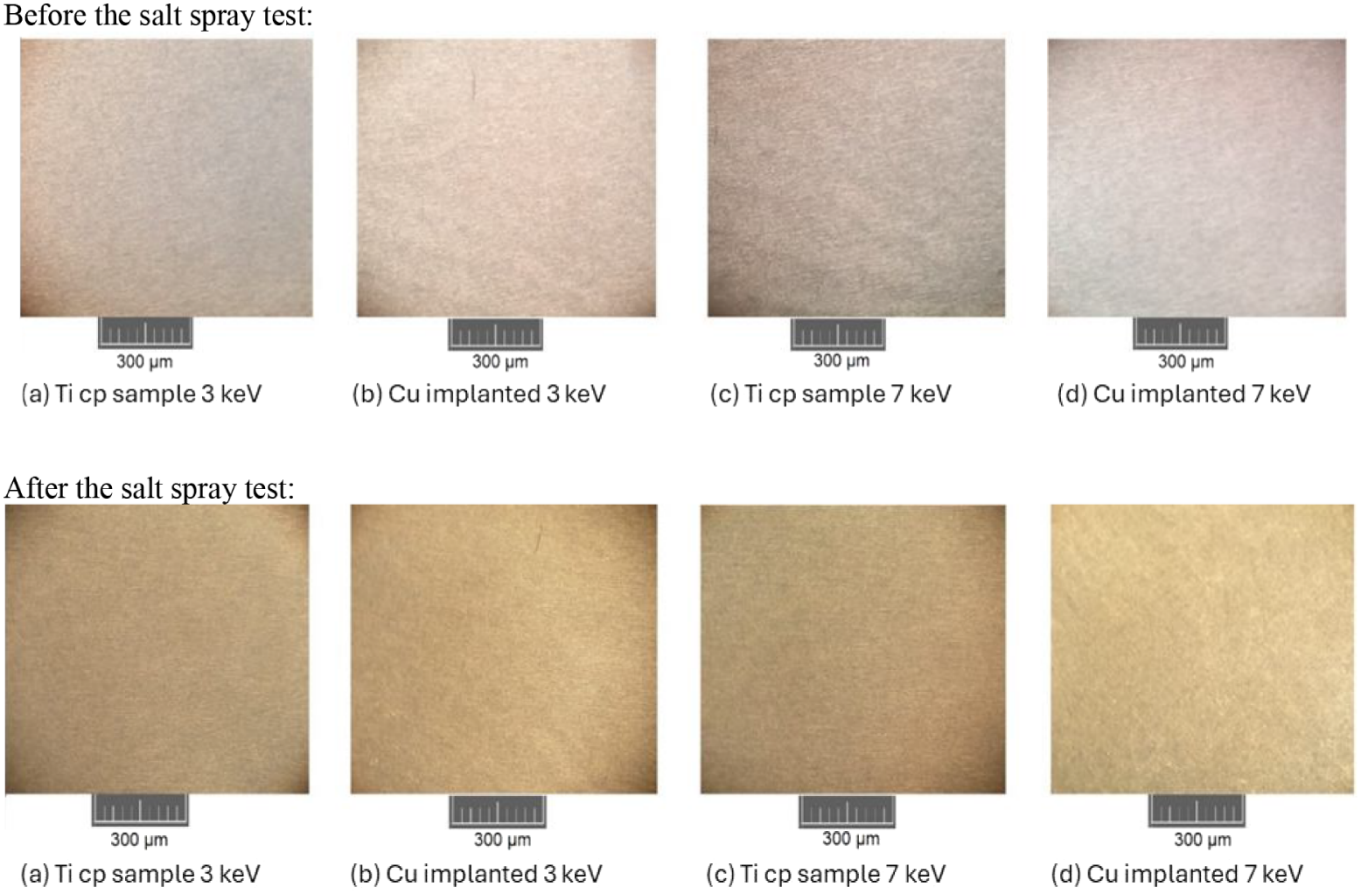
Microscopic analysis before and after the salt spray test.

After salt-spray exposure, XRF analyses were performed again on the Cu-implanted samples to evaluate the possible loss of copper due to corrosion or leaching. The results showed Cu concentrations of 0.27 wt % for the 3 keV condition and 0.29 wt % for the 7 keV condition, values that are practically identical to those measured prior to testing. These findings indicate that no significant copper loss occurred during salt-spray exposure, confirming the good chemical stability of the implanted layer and supporting the conclusion that the IPD process produced a firmly incorporated subsurface modification resistant to leaching.

### Biological Tests

The antibacterial activity of the Cu-implanted titanium samples was assessed by using both qualitative and quantitative assays against *Staphylococcus aureus* ATCC 25923.

In the qualitative agar diffusion test, none of the samples exhibited inhibition halos around their perimeters after 24 h of incubation. This result indicates that the implanted copper did not diffuse into the culture medium and, therefore, did not generate an inhibition zone characteristic of bactericidal materials. Bacterial growth was observed both on the agar surface and directly over the materials, suggesting that the antimicrobial effect was negligible or limited to weak bacteriostatic behavior.

These findings were corroborated by quantitative turbidity measurements (OD_60_) performed on liquid cultures after 24 h of incubation. The optical density values obtained for the Cu-implanted samples (3 keV and 7 keV) were statistically similar to those of the unmodified titanium control, confirming the absence of a significant reduction in bacterial growth. These results are shown in Figure 6.

**Figure 6:**
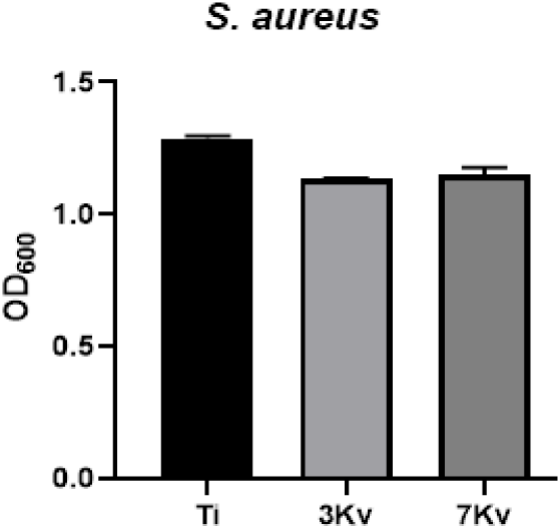
Quantitative antibacterial analysis of Ti- and Cu-implanted Ti samples (3 keV and 7 keV) against *Staphylococcus aureus* ATCC 25923.

The fluorescence microscopy analysis using the LIVE/DEAD BacLight kit further supported these results. As shown in Figure 7, the bacterial suspensions exhibited a predominance of green-stained (viable) cells, with only a few red-stained (nonviable) cells. This pattern was consistent across all conditions, including the control, indicating that neither of the implanted surfaces induced measurable membrane damage or cell death.

**Figure 7:**
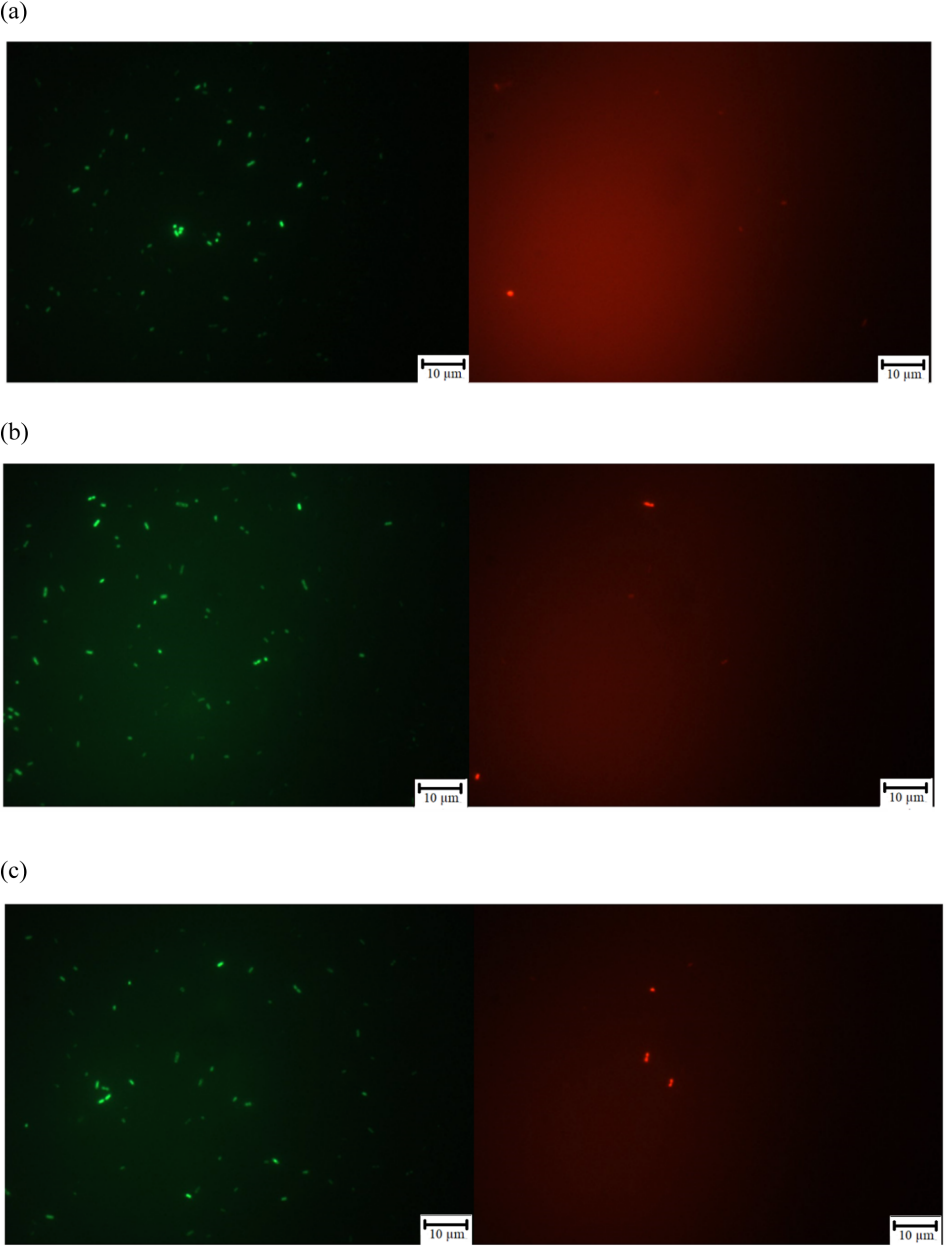
Fluorescence test for *S. aureus* grown in agar medium and exposed to treated or untreated surfaces. Green: living bacteria; red: dead bacteria. (a) Substrate without implantation; (b) implantation with 3 keV; (c) implantation with 7 keV.

The lack of bactericidal or bacteriostatic responses can be attributed to two main factors. First, XRF analysis showed that the total copper content was below 0.5 wt %, a concentration likely insufficient to achieve antibacterial effectiveness. According to Liu et al., the Cu content should be at least 5 wt % to obtain strong antibacterial activity.^34^ Second, SRIM simulations demonstrated that copper ions were incorporated at subsurface depths (≈4 nm for 3 keV and ≈6 nm for 7 keV), below the active contact layer. Under such conditions, the implanted Cu cannot directly interact with bacterial cells or release ions into the surrounding medium.^35^ Therefore, the observed absence of inhibition halos or reduction in viable cells directly corroborates the implantation profile predicted by simulation, validating that the chosen ion energies (3–7 keV) are below the threshold required for surface-active antimicrobial behavior.

## Conclusion

Qualitative and quantitative analyses confirmed the successful implantation of copper into titanium, showing that despite the different implantation energies, the overall Cu content remained similar in both samples. SRIM simulations indicated that higher implantation energy resulted in deeper Cu ion penetration, whereas lower energy concentrated ions closer to the surface. Corrosion tests demonstrated that the incorporation of copper did not compromise the intrinsic corrosion resistance of titanium. Biological evaluations revealed no inhibition halos or significant reduction in bacterial viability, indicating weak to no bacteriostatic activity. This result is attributed to the low Cu concentration (<0.5 wt %) and the subsurface location of the implanted ions, which prevent effective interaction with bacterial cells. Although no antimicrobial effect was observed, these findings are valuable for establishing the energy range in which copper remains confined within the subsurface region without altering titanium’s surface integrity. This understanding provides a solid foundation for optimizing future implantation parameters to achieve balanced mechanical, corrosion, and antibacterial performance
